# Social support networks and well-being of disabled veterans: the dual roles of institutional support and peer trust

**DOI:** 10.3389/fpsyg.2025.1654845

**Published:** 2025-08-22

**Authors:** Qiuyue Zhang, Yi Wang, Yili Lin, Yu Cao, XiaoBin Guan

**Affiliations:** ^1^School of Economics and Management, Beijing University of Technology, Beijing, China; ^2^School of Peace and Development, Renmin University of China, Beijing, China; ^3^Center for Applied Statistics, Renmin University of China, Beijing, China

**Keywords:** disabled veterans, social support networks, subjective well-being, institutional support, peer trust

## Abstract

**Introduction:**

Existing studies have consistently demonstrated a positive association between social capital and subjective well-being; however, systematic evidence on this relationship among disabled veterans remains limited. This study investigates how structural social capital—captured by the breadth of social support networks—affects the subjective well-being of disabled veterans in China. It further examines the mediating roles of perceived effectiveness of government assistance (institutional resource utilization) and comrade trust (relational social capital), as well as the moderating role of policy awareness in shaping these relationships.

**Methods:**

A stratified sampling strategy was employed to collect survey data from 472 disabled veterans across multiple regions in China. Structural social capital was measured through the size and diversity of respondents’ social support networks. Ordered Logit regression models were used to estimate the effects, and mediation and moderation analyses were conducted to assess underlying mechanisms.

**Results:**

First, broader social support networks were found to be significantly and positively associated with higher levels of subjective well-being. Second, both perceived government assistance effectiveness and comrade trust significantly mediated this relationship, reflecting the dual embeddedness of institutional and relational resources. Third, policy awareness moderated the association, indicating a marginal substitution effect between formal and informal capital. Finally, the positive effect of structural social capital was more pronounced among older veterans and those with less severe disabilities.

**Discussion:**

The findings highlight a synergistic mechanism between institutional services and social network resources in enhancing disabled veterans’ well-being. Policy implications include strengthening social support networks, improving institutional responsiveness, and increasing policy awareness, while tailoring interventions by age and disability severity. Such measures can more effectively transform social capital into psychological well-being.

## 1 Introduction

Subjective well-being, as a key indicator of individual psychological welfare, has become a central topic in social psychology and social policy research. Veterans, especially those disabled due to service, constitute a special group shaped by a highly institutionalized environment and undergoing drastic social transitions. Their subjective well-being not only reflects their individual welfare but also indicates the effectiveness of national institutional protections and social integration mechanisms. Compared to the general population, veterans who suffered trauma during service often face more complex physical and psychological challenges as well as social adaptation difficulties, exhibiting greater vulnerability in well-being (Paat et al., 2025). Although existing studies acknowledge the positive effects of policy interventions on veterans’ living conditions, the psychological welfare of disabled veterans remains insufficiently examined. Against this background, this study raises the core research question: How do social support networks affect the subjective well-being of disabled veterans?

Although the current veteran support system has gradually improved its structural support measures, issues remain in institutional coordination and implementation. On one hand, while existing veteran resettlement policies have established a multi-agency collaborative framework, there is still a lack of institutional linkage and overall coordination among military disability insurance, compensation and preferential treatment, and social welfare systems (Yue and Zheng, 2023). On the other hand, some policies lack stable and clear legal foundations, and inconsistent enforcement mechanisms have led to a “policy-without-access” dilemma at the grassroots level (Yang et al., 2023). Under this context, disabled veterans face restricted access to resources, further weakening their social integration capacity and psychological resilience. When structural support is constrained, informal social resources—social capital—may play a critical role in compensating for institutional gaps and enhancing psychological well-being (Kyambade et al., 2024). Compared to formal institutional guarantees, social capital offers irreplaceable functions in emotional comfort, information sharing, and trust building (Nutakor et al., 2023). Particularly for disabled veterans marginalized by institutional deficiencies and limited resources, support derived from social networks may become a key pathway to sustaining their subjective well-being (Gettings et al., 2022; Zhou et al., 2021; Rhodes et al., 2024).

Although the relationship between social capital and well-being has received widespread attention, existing research still presents certain limitations in terms of target populations and mechanism exploration. First, most studies focus on the general population, with insufficient attention to highly institutionalized and potentially marginalized groups, such as disabled veterans. This group bears the dual burden of institutional role transition and the challenges of physical and psychological trauma, along with social exclusion. Their subjective well-being is thus more susceptible to variations in resource access and the degree of social embeddedness. Second, current research predominantly employs regression models to examine the correlation between social capital and well-being, with limited exploration of potential mediating pathways or moderating mechanisms. In particular, few studies systematically identify the dual roles of institutional and relational resources. Such a “black box” approach constrains our understanding of how social capital influences the psychological well-being of vulnerable groups and diminishes the policy relevance of related findings.

At the theoretical level, there is still debate regarding the pathways through which social capital influences individual well-being. While existing studies have distinguished between the functions of structural and relational social capital, a unified framework explaining how these two forms jointly shape psychological well-being remains underdeveloped. In particular, under conditions of unequal access to institutional resources and high dependence on informal trust networks, it remains unclear whether structural social capital operates through a dual-pathway mechanism—via institutional resources such as government support and relational resources such as trust among fellow veterans. Both theoretical clarification and empirical evidence are lacking in this regard.

Recent studies have begun to explore individual well-being through the dual dimensions of institutional embeddedness and social interaction, suggesting that formal institutional resources and informal trust relations jointly constitute key sources of social capital and play a critical role in enhancing well-being (Rodríguez-Pose and Von Berlepsch, 2014). On one hand, structural social capital—such as social networks and institutional trust—can expand access to resources, strengthen cooperative expectations, and improve income levels and life satisfaction. On the other hand, social support contributes positively to mental health by enhancing psychological resilience, filling emotional gaps, alleviating depressive symptoms, and improving overall mental states (Cohen and Wills, 1985). Furthermore, social support has been found to reduce family stress among disabled veterans (Frank et al., 2022). Although relevant theoretical and empirical studies are growing, the application of this social capital framework in veteran studies remains limited. Therefore, it is necessary to incorporate both institutional and relational social capital into an integrated analytical perspective, identify the underlying mechanisms through which structural social capital affects well-being, and further examine how moderating factors shape the boundaries of its effects.

Building on this foundation, the present study places the mechanism through which structural social capital influences subjective well-being at the center of analysis. It focuses on the dual mediating pathways of institutional and relational resources and examines the moderating effect of policy awareness. This approach aims to address two major gaps in current social capital research: the limited identification of causal mechanisms and the lack of attention to group heterogeneity. Specifically, this study seeks to answer three key questions: First, does the structural social capital of veterans—measured by social support networks—affect their subjective well-being through two mediating mechanisms: service feedback from government assistance (institutional resources) and trust among fellow veterans (relational resources)? Second, does policy awareness exert a significant moderating effect on this mechanism, potentially altering the effectiveness of access to social capital resources? Third, do these mechanisms vary systematically across different subgroups of veterans? By constructing a mediation model and a contextual moderation framework, this study aims to uncover the internal logic by which social capital is transformed into psychological well-being within the context of institutional embeddedness.

To address the above questions, this study draws on data from 472 disabled veterans surveyed in the 2023 National Veterans Survey. It employs an ordered logit model and a mediation–moderation analysis strategy to construct a comprehensive empirical framework that integrates direct, mediating, and moderating effects. The study first measures the breadth of social support networks to assess the objective coverage of structural social capital. It then introduces two mediating variables: perceived effectiveness of government assistance and trust in fellow veterans, to examine how these channels transmit the effects of social capital on subjective well-being. Policy awareness is incorporated as a moderating variable to analyze its influence on the strength and boundary conditions of these mediating pathways. Data were collected using stratified sampling to ensure representativeness. Analytically, the study combines a four-step mediation test with interaction term regression analysis. This research makes three key contributions: First, it focuses on disabled veterans under institutional transition, addressing a gap in the literature; Second, it identifies both institutional and relational mediating pathways to clarify the mechanisms through which social capital affects well-being; Third, it offers evidence-based insights for developing a tiered, targeted psychological support system aligned with differentiated needs and mechanism-based interventions.

## 2 Literature review and hypothesis

The concept of social capital was first introduced by French sociologist Pierre Bourdieu, who defined it as a set of actual or potential resources linked to institutionalized relationships and embedded within social networks (Bourdieu, 2011). From a political science perspective, Putnam et al. (1994) conceptualized social capital as an organic whole composed of features inherent in social structure—such as social trust, norms of reciprocity, and civic engagement—which enhance collective cooperation and improve the efficiency of social functioning. Building on Putnam’s work, Scheufele and Shah (2000) further categorized social capital into three dimensions: structural, cognitive, and relational embeddedness. Structural embeddedness focuses on impersonal linkages between individuals and organizations; cognitive embeddedness involves shared language, values, and meaning systems within a social network; and relational embeddedness highlights interpersonal relationships formed through long-term interactions. Unlike human capital, which emphasizes individual abilities, some scholars define social capital as a relational resource derived from social ties and networks that can only be accessed and mobilized through purposeful actions (Bhandari and Yasunobu, 2009).

Subjective well-being is shaped not only by material satisfaction but also by the degree of social connectedness. A broader social network enhances individuals’ sense of recognition and belonging, thereby increasing their overall happiness (Li et al., 2020). Numerous studies have confirmed the link between individual well-being and social support networks. Portela et al. (2013) found that social networks, interpersonal trust, and institutional trust are all significantly and positively associated with subjective well-being. Research on rural migrant workers in China further reveals that social capital has a substantial impact on their well-being; the wider their social network, the higher their reported levels of happiness (Wu et al., 2017). The breadth of social networks, social trust, and institutional trust together constitute key pathways through which social capital enhances subjective well-being (Rodríguez-Pose and Von Berlepsch, 2014). Broad and stable networks facilitate the flow of information and emotional bonds, foster mutual trust and reciprocity, and reinforce consensus. As individuals engage more frequently in social interactions, interpersonal trust tends to increase. In turn, this trust and reciprocity strengthen the resilience and functionality of social networks, promote the accumulation of social capital, and ultimately improve the efficiency of collective action (Claridge, 2018; Page-Tan, 2021).

Social capital can enhance individuals’ subjective well-being by improving both mental health and economic benefits. In terms of psychological well-being, social networks provide a foundation for emotional communication and social support, which help improve mental health and thereby indirectly increase well-being (Ye and Zhang, 2021). Trust bonds within strong ties—such as kinship and neighborhood relations—can also enhance subjective well-being by strengthening psychological resilience (Brajša-Žganec et al., 2024). Specifically, such trust reduces anxiety and enhances the perception of social support, which positively contributes to the experience of happiness (Yu, 2017). Higher perceived social support is associated with better mental health outcomes. Additional studies show that social support networks significantly improve psychological adaptability among left-behind children through enhanced resilience and emotional compensation (Fan and Fan, 2021), improve the mental health of left-behind elderly, reduce depressive symptoms among older adults (Visaria et al., 2020), and promote mental well-being among young migrant populations (Zhou et al., 2022). Regarding economic benefits, two core components of social capital—network size and breadth of trust—enhance access to resources and strengthen expectations for cooperation, thus significantly contributing to income growth and greater well-being. Xie and Wang (2016) categorize rural household social capital into two types: localized social capital, based on kinship and neighborhood trust; and delocalized social capital, shaped by occupational mobility and spatial transitions. Their findings suggest that delocalized social capital has a more pronounced positive effect on income growth. Social capital thus enables individuals to gain advantages in employment opportunities and income generation, improving economic status and, in turn, enhancing perceptions of well-being.

For disabled veterans, the role of social capital is particularly critical. Due to physical limitations and challenges associated with identity transitions, this group often faces barriers to social integration, including limited access to information, restricted employment opportunities, and social isolation. As a result, they rely more heavily on the support and resources provided by social networks (Brucker, 2015; Mithen et al., 2015). In addition, having lived in a highly institutionalized military environment, veterans often face significant difficulties in reconstructing interpersonal relationships and social ties after discharge. The regeneration of social networks plays a key role in their social reintegration and psychological adjustment (Angel et al., 2018). Therefore, the breadth of social networks not only affects their access to emotional and material support but also significantly influences their ability to successfully transition from the identity of a “soldier” to that of a “civilian.” In this context, broader social networks increase the likelihood that disabled veterans will experience social recognition and a sense of belonging, thereby enhancing their subjective well-being.

Therefore, the following hypothesis is proposed:

*H1: Greater breadth of social support networks is associated with higher levels of subjective well-being among disabled veterans.*

From a functionalist perspective, Coleman (1990a,b) conceptualized social capital as social resources and norms generated through interpersonal interactions, social networks, and institutional arrangements. These resources promote goal attainment by enhancing trust and cooperation among actors. Building upon this foundation, Krishna (2002) further distinguished social capital into two dimensions: institutional social capital and relational social capital. Institutional social capital refers to individuals’ interactions with formal institutions and organizations, encompassing regulatory frameworks, policy information, public service provision, and trust in formal systems. It primarily reduces transaction costs by offering structured information and resources through institutional transparency and stability (Putnam et al., 1994). In contrast, relational social capital emphasizes informal social networks developed through long-term interpersonal interactions—such as kinship, friendship, and community ties—characterized by trust, reciprocity, and social identification. These relational resources mainly function as emotional support and informal channels of information (Nahapiet and Ghoshal, 1998; Lin, 2001). In the context of this study, institutional social capital is operationalized as veterans’ perceived effectiveness of government assistance, while relational social capital is represented by the psychological support derived from mutual trust among comrades.

Institutional trust and the quality of public service provision, as key dimensions of institutional social capital, influence individuals’ subjective well-being by enhancing their perceptions of social justice and security. As previously noted, social capital generally takes two forms: institutional social capital, which includes structural elements such as rules, procedures, and organizations; and relational social capital, which refers to values, attitudes, and beliefs formed through social interactions. The interplay between the structural rigidity of institutional elements and the cognitive flexibility of relational elements jointly enhances the effectiveness of collective action. In a quasi-experimental study, Schneider et al. (1997) examined how differences in parental school choice affected parental behavior, finding that well-designed institutional arrangements can increase individual participation in activities that foster social capital. Their findings suggest that institutional arrangements concerning regional public goods can shape the development of social capital.

Social networks facilitate more effective information transmission, thereby reducing information costs and, to some extent, enhancing the effectiveness of policy assistance. Government support, through the allocation of institutional resources, stimulates social vitality, fosters the development of social capital, and improves the provision of public services, ultimately increasing public satisfaction (Shen et al., 2025). From the perspective of social exchange theory, social interactions and relationships are understood as processes of reciprocal benefit, where individuals and groups engage in exchanges within socially embedded contexts to obtain desired resources. According to this theory, social actors build reciprocal relationships through exchange, receiving valuable returns, and the foundation of these social contracts lies in the generation and maintenance of trust (Ahmad et al., 2023). When individuals receive effective support from the government, they experience psychological affirmation and a sense of reciprocity, which in turn enhances their overall well-being.

The positive impact of government assistance on the well-being of disabled veterans operates primarily through two key pathways. First, it provides economic security. By implementing subsidy policies, the government offers financial support and welfare compensation aimed at improving the living conditions and overall well-being of disabled veterans. These subsidies are regarded as a direct means of meeting basic needs, alleviating financial stress, and enhancing quality of life (Umucu et al., 2024). Second, it promotes social integration. The well-being of disabled veterans is closely linked to their ability to adapt to civilian life. The government actively implements employment support policies, offering vocational training, job opportunities, and career development assistance. Gaining access to stable employment and income enables veterans to realize self-worth and attain social recognition, thereby enhancing their subjective well-being (Banta, 1945).

Therefore, the following hypothesis is proposed:

*H2a: The effectiveness of government assistance mediates the relationship between the breadth of social support networks and subjective well-being, reflecting the transmission mechanism of institutional resources.*

The essential function of social support lies in its dual role—through social networks and social capital—in regulating emotional experiences. It enhances the generation of positive emotions while buffering negative psychological stimuli, ultimately improving subjective well-being. Within the context of China’s collectivist culture, the deeply embedded hierarchical relational structure permeates daily life, granting social capital a unique capacity to empower individual action. According to Coleman’s (1990a,b) theoretical framework, trust mechanisms are central to the allocation of resources and the maintenance of social networks. He defines social capital as a form of capital embedded in social interactions, composed of various elements that individuals can mobilize and transfer to others in exchange for control over valuable resources. Wang and Hu (2021) found that trust among group members is positively associated with cooperation efficiency—higher levels of trust lead to faster formation of collaborative relationships and lower friction costs during interaction. Individuals with strong relational social capital tend to engage in more frequent and longer-lasting interactions characterized by higher levels of mutual trust and reciprocity. Such deeply embedded relational networks are often formed among individuals with similar life settings, work environments, and personal backgrounds, typically sharing comparable cultural and cognitive characteristics (Liu et al., 2014).

Peer trust, as a form of high in-group trust, is a key component of affective social capital. It promotes well-being by providing emotional support and a sense of security. As one of the primary relationships within the social network of disabled veterans, peer relationships are built on shared experiences in highly similar environments, leading to strong, stable bonds of trust. This form of strong-tie support can buffer the negative effects of psychological stress and social exclusion, enhance positive psychological states, and ultimately improve overall well-being.

For veterans, especially those with disabilities, the camaraderie accumulated during military service can be transformed into an “extended relational capital” after discharge, characterized by both the emotional depth of strong ties and the resource accessibility of semi-strong ties. Specifically, in terms of the relationship between comrade trust and well-being, Drebing et al. (2018) argued that veterans can enhance community reintegration through “peer support groups,” gaining substantial social support and psychological comfort during this process, which facilitates identity recognition and emotional regulation, ultimately improving overall well-being and social integration. From a social capital perspective, Albertson et al. (2015) further demonstrated that “comradeship” and mutual trust among veterans serve as crucial pathways for psychological recovery and social reconstruction, particularly for veterans with histories of criminal behavior or substance abuse. Such camaraderie extends the sense of military identity, provides resource exchange and emotional support, and has a positive impact on individual well-being.

From the perspective of resource acquisition, comrade trust also expands the scope and influence of social support networks. A study on the accumulation and impact of social capital found that the majority of veterans reported receiving entrepreneurial advice, financial support, or daily assistance from their comrades, and most believed that the camaraderie and mutual assistance developed during service formed the foundation of their entrepreneurial success (Gao, 2021). Similarly, Guerrero et al. (2021) supported this view, noting that identity-based trust among veterans enhances the effectiveness of social support, with mutual aid, resource sharing, and emotional companionship significantly improving veterans’ social adaptability and well-being, while also aiding recovery from substance use disorders.

Therefore, the following hypothesis is proposed:

*H2b: Peer trust mediates the relationship between the breadth of social support networks and subjective well-being, reflecting the transmission mechanism of relational resources.*

An individual’s awareness of policies is closely linked to government transparency. Government transparency reflects both the extent to which authorities proactively disclose information and the feasibility for citizens to request such information. When transparency is high, the information gap between citizens and the government narrows, reducing the cost of accessing policy-related information and enhancing individuals’ sense of empowerment. Moreover, it enables citizens to exercise their rights more effectively and participate in community governance, thereby strengthening their perception of social fairness (Zheng et al., 2022). According to the resource substitution theory, institutional information and social support networks can serve as partial substitutes in meeting individual well-being needs. When policy awareness is high, institutional resources compensate for the reliance on network-based resources, leading to diminishing marginal effects.

As a core dimension of institutional informational capital, policy awareness weakens the marginal effect of social support networks through two primary pathways. From a resource perspective, policy information is characterized by standardization, systematization, and authority, enabling it to address institutional needs more efficiently and partially substitute for the informal information transmission within social networks. According to the capability-opportunity interaction model (Sen, 1993), individuals’ well-being outcomes are jointly determined by their “capabilities” such as the ability to utilize social networks, and “opportunities” such as access to policy information. These two factors interact and can serve as functional substitutes. Lu and Yang (2013) mathematically demonstrated the impact of fiscal transparency on subjective well-being. Similarly, Liang (2017) argued that the transparency of relevant policies—the extent to which local governments disclose information—is a subjective behavior of local authorities, while satisfaction with public services reflects residents’ subjective evaluation of the services and goods provided. The levels of both influence public trust in local government and, consequently, affect residents’ subjective well-being.

The moderating effect of policy awareness operates through three key mechanisms. First, it reduces information costs. Policy awareness can be viewed as a form of “informational capital” which influences the marginal utility of social capital. Higher policy transparency lowers the transaction costs of accessing information. Second, it mitigates risk. The official nature of policy information reduces the risk of misinformation. When facing major decisions, individuals with higher policy awareness are more likely to rely on official information rather than interpersonal advice. Third, it fosters psychological empowerment. Policy awareness enhances perceived self-efficacy and fairness, thereby indirectly improving subjective well-being. Furthermore, policy awareness primarily substitutes for the instrumental functions of social networks—such as information transmission—while emotional support remains dependent on strong-tie networks.

For disabled veterans, physical limitations and restricted access to social information often lead to a greater reliance on formal institutional channels to obtain welfare and services. In this context, *policy awareness* functions as a form of *institutional informational capital*, with its level directly affecting an individual’s access to and utilization of official resources (Akaeda, 2025; Fleming et al., 2016). When government information is transparent and timely, such institutional knowledge can meet veterans’ instrumental needs in a standardized and authoritative manner, thereby reducing their dependence on informal networks and producing a *substitution effect* between formal and informal resources (Hanchate et al., 2018; Finkelstein and Notowidigdo, 2019). This substitution mechanism is particularly relevant for veterans with limited mobility, as higher levels of policy awareness can lower the cost of information search and enhance self-efficacy (Drum and Ditsch, 2025), ultimately weakening the marginal contribution of social support networks to subjective well-being.

Therefore, the following hypothesis is proposed:

*H3: Policy awareness moderates the relationship between the breadth of social support networks and subjective well-being. Specifically, when policy awareness is high, the positive effect of social support networks on subjective well-being is weakened, reflecting a marginal substitution effect.*

## 3 Research design

### 3.1 Data source

The data used in this study were derived from a 2023 survey of veterans conducted in nine major troop-contributing provinces in China: Jilin, Sichuan, Shandong, Guangdong, Guangxi, Jiangsu, Zhejiang, Fujian, and Liaoning. These provinces are considered representative and can, to a certain extent, reflect the overall characteristics of the national veteran population. Given the significant regional disparities in the distribution of veterans across China, this study employed geographically stratified sampling to ensure the representativeness and generalizability of the sample. Specifically, provinces served as the first-level strata, within which the sample was proportionally allocated across prefecture-level administrative units based on the registered number of veterans. This approach minimizes underrepresentation of provinces with smaller veteran populations, reduces overall sampling variance, and enhances statistical power. Based on field interviews with disabled veterans in Shandong Province, the study found that social support plays a crucial role in shaping their subjective well-being. To further explore this relationship, the research team developed and implemented a structured questionnaire covering service history, individual agency, and structural factors to systematically collect micro-level data (see Appendix I for the questionnaire).

During data collection and processing, the study strictly adhered to ethical review standards and institutional regulations to ensure respondents’ rights to informed consent, privacy, and voluntary participation. To improve the quality and representativeness of the sample, the following data cleaning procedures were applied: First, questionnaires missing key information (e.g., discharge year) were removed. Second, records with logical inconsistencies (e.g., discharge occurring before enlistment, or a disability rating of zero) were excluded. Third, according to China’s official criteria for veteran status, cases with less than two years of service were removed. Finally, for continuous variables, extreme values were winsorized at the 1st and 99th percentiles to reduce their influence on the analysis. After applying these procedures, a total of 472 valid responses were retained.

### 3.2 Model construction

To examine the effect of social support network breadth on the subjective well-being of disabled veterans, this study treats well-being as an ordinal categorical dependent variable, classified into three levels based on self-assessed evaluations: very poor (coded as 1), average (coded as 2), and very good (coded as 3). Given that subjective well-being reflects a ranked order without equidistant intervals between categories, the use of OLS or Poisson models may result in biased estimates. In contrast, the ordered Logit (ologit) model is specifically designed for such ordinal dependent variables and provides consistent estimates under the assumption of proportional odds. Therefore, this study employs an ordered Logit regression model and further controls for province-level fixed effects to account for potential regional heterogeneity.

The baseline model is specified as follows (Equation 1):


(1) $$\begin{array}{l} \text{Logit}[P(\text{Well}-\text{being}\geq K|X)]=\beta_{0}+\beta_{1}{\text{Network}}_{i}\\ +\sum \beta_{2}{\text{Control}}_{i}+\delta_{s}+\varepsilon_{i}\; \end{array}$$

Here, *network* denotes the breadth of social support networks (measured as the logarithm of total mobile contacts), and *control* represents the set of control variables, including *party_member*, *service_time*, *income*, *marriage*, *education*, *branch*, *rural*, *age*, *male*. $\delta_{s}$ denotes province fixed effects, and $\varepsilon_{i}$ is the random error term.

To examine the underlying mechanisms through which social support network breadth (*network*) affects subjective well-being (*well-being*), this study further constructs Equations 2–4, which, together with the baseline Equation 1, form a four-step mediation analysis framework. The mediator variable (*mediator*) represents either the individual’s perceived effectiveness of institutional support (*effective*) or peer trust (*trust*), capturing the transmission paths of institutional and relational social capital, respectively. All models control for province fixed effects $\delta_{s}$ to account for unobserved regional heterogeneity.

Equation 2 tests the effect of the independent variable *network* on the mediating variable *effective* or *trust*:


(2) $${\text{Mediator}}_{i}=\beta_{0}+\beta_{1}{\text{Network}}_{i}+\sum \beta_{2}{\text{Control}}_{i}+\delta_{s}+\varepsilon_{i}$$

Equation 3 examines the effect of the mediating variable *effective* or *trust* on *Well-being*:


(3) $$\begin{array}{l} \text{Logit}[P(\text{Well}-\text{being}\geq K|X)]=\beta_{0}+\beta_{1}{\text{Mediator}}_{i}\\ +\sum \beta_{2}{\text{Control}}_{i}+\delta_{s}+\varepsilon_{i} \end{array}$$

Equation 4 includes both the independent variable and the mediating variable to test whether a mediation effect exists:


$$\text{Logit}[P(\text{Well}-\text{being}\geq K|X)]=\beta_{0}+\beta_{1}{\text{Network}}_{i}+\beta_{2}{\text{Mediator}}_{i}+$$

(4) $$\sum \beta_{3}{\text{Control}}_{i}+\delta_{s}+\varepsilon_{i}$$

The identification of a mediation effect must meet the following conditions: In Equation 1, a significant $\beta_{1}$ indicates that *network* has a significant total effect on *Well-being*. In Equation 2, a significant $\beta_{1}$ suggests that *network* significantly affects the mediator variable (*effective* or *trust*). In Equation 3, a significant $\beta_{1}$ implies that the mediator variable has a significant effect on *Well-being*. In Equation 4, if the coefficient $\beta_{1}$ of *network* decreases in magnitude compared to that in Equation 1 but remains significant, it indicates the presence of a partial mediation effect. If the coefficient $\beta_{1}$ of *network* becomes insignificant (or approaches zero) compared to that in Equation 1, it indicates a full mediation effect.

To further examine the moderating effect of policy awareness on the relationship between social support networks and subjective well-being, the following ordered logit regression model is constructed the following ordered logit regression model is constructed (Equation 5):


(5) $$\begin{array}{l} \text{Logit}[P(\text{Well}-\text{being}\geq \mathrm{KX})]=\beta_{0}+\beta_{1}{\text{Network}}_{i}+\beta_{2}{\text{Awareness}}_{i}\\ +\beta_{3}{\text{Network}}_{i}*{\text{Awareness}}_{i}\\ +\sum \beta_{4}{\text{Control}}_{i}+\delta_{s}+\varepsilon_{i} \end{array}$$

Here, awarenessdenotes policy awareness, and network∗awarenessrepresents the interaction term between social support networks and policy awareness. A significantly negative coefficient for $\beta_{3}$ indicates that higher policy awareness weakens the positive effect of social support networks on well-being, suggesting a marginal substitution effect.

The definitions and measurement methods of all variables are detailed in Table 1.

**Table 1:** Variable definition and measurement.

Variable name	Variable type	Measurement and coding description
Well-being	Ordered categorical variable	Self-rated overall life and work status: Very Poor = 1; Average = 2; Very Good = 3.
Network	Continuous Variable (log-transformed)	Breadth of social connections, Measured as the natural logarithm of the total number of contacts in the respondent’s mobile phone plus one.
Effective	Ordered categorical variable	Measuring the respondent’s evaluation of the effectiveness of the veterans affairs department in addressing their requests. It is coded as: very poor = 1; average = 2; very good = 3.
Trust	Continuous variable	Reflecting the respondent’s overall trust in the veteran community, measured on a linear 0–10 scale, where 0 = completely distrust and 10 = completely trust.
Awareness	Ordered categorical variable	Capturing the respondent’s level of understanding of veteran-related policies. It is coded as: completely unaware = 1; somewhat aware = 2; very aware = 3.
Party_member	Binary variable	Chinese Communist Party membership: Yes = 1; No = 0.
Service_time	Continuous variable	Years of military service, Actual number of years served in the military.
Income	Continuous variable (log-transformed)	Natural logarithm of total personal income for the year 2022.
Marriage	Ordered categorical variable	Marital status: Married or Widowed = 1; Divorced = 2; Unmarried = 3.
Education	Ordered categorical variable	Education level: Low (primary school and below, junior high school, vocational school, technical school) = 1; Medium (high school, junior college) = 2; High (bachelor’s degree and above) = 3.
Branch	Ordered categorical variable	Branch of military service: Other (e.g., Rocket Force, Strategic Support Force, nuclear-related units) = 0; Navy = 1; Army = 2; Air Force = 3; Armed Police Force = 4.
Rural	Binary variable	Type of household registration, Urban = 0; Rural = 1.
Age	Continuous variable (log-transformed)	Natural logarithm of actual age in 2023.
Male	Binary variable	Gender: Male = 1; Female = 0.

## 4 Empirical analysis

### 4.1 Data description

Building on the research design and variable measurement, this section tests the theoretical hypotheses using descriptive statistics and regression models. Based on 472 valid samples, the analysis first presents the distributional characteristics of key variables. It then uses baseline regressions to examine the direct effect of social support networks on subjective well-being, followed by mediation models to explore the transmission mechanisms of institutional support and comrade trust. Finally, moderation and heterogeneity analyses are conducted to identify the boundary conditions of these effects.

According to the descriptive statistics in Table 2, the average subjective well-being score among the 472 disabled veteran respondents is 2.252, indicating a moderately high level, with a standard deviation of 0.637, suggesting relatively small variation across individuals. The average breadth of social support networks is 4.258, corresponding to approximately 71 mobile contacts, with a standard deviation of 2.029, indicating substantial variation in social connectedness. Party members account for 80.7% of the sample, with an average of 9.125 years of military service. The mean logarithm of annual personal income is 10.871, equivalent to approximately 62,000 yuan. Most respondents are married, with education levels concentrated between low and medium levels. The majority served in the Army, and about 47.6% hold rural household registration. The mean log-transformed age is 3.818, corresponding to approximately 45 years old, and males account for 97.5% of the sample.

**Table 2:** Descriptive statistics results.

Stats	N	Mean	SD	Min	Max	p50
Well-being	472	2.252	0.637	1.000	3.000	2.000
Network	472	4.258	2.029	0.000	7.352	4.796
Party_member	472	0.807	0.395	0.000	1.000	1.000
Service_time	472	9.125	5.674	3.000	25.000	7.000
Income	472	10.871	0.642	8.006	12.206	10.820
Marriage	472	1.248	0.625	1.000	3.000	1.000
Education	472	1.727	0.790	1.000	3.000	2.000
Branch	472	2.136	1.136	0.000	4.000	2.000
Rural	472	0.477	0.500	0.000	1.000	0.000
Age	472	3.818	0.301	3.219	4.394	3.807
Male	472	0.975	0.158	0.000	1.000	1.000

### 4.2 Baseline regression

The regression results in Table 3 indicate that the breadth of social support networks (*network*) has a significant positive effect on the subjective well-being of disabled veterans. In the baseline model without control variables (Column 1), the regression coefficient for network is 0.203 (*p* < 0.01). After controlling for a range of individual characteristics (Column 2), the coefficient remains positive and significant at 0.183 (*p* < 0.01). These results suggest that disabled veterans with broader social support networks report higher levels of subjective well-being, thus supporting Hypothesis H1.

**Table 3:** Baseline regression results of social support network breadth on subjective well-being.

Variables	(1)			(2)		
	Well-being			Well-being		
	Coef.	SE	p	Coef.	SE	p
Network	0.203***	(0.048)	0.000	0.183***	(0.051)	0.000
Party_member				0.192	(0.265)	0.468
Service_time				0.003	(0.021)	0.866
Income				0.348**	(0.169)	0.039
Marital status (reference group: married)						
2.Marriage				−0.391	(0.457)	0.392
3.Marriage				−0.483	(0.374)	0.196
Education level (reference group: low education)						
2.Education				0.514**	(0.258)	0.046
3.Education				−0.204	(0.309)	0.509
Military branch (reference group: other)						
1.Branch				−0.379	(0.418)	0.365
2.Branch				−0.210	(0.336)	0.531
3.Branch				−0.682	(0.456)	0.135
4.Branch				0.322	(0.389)	0.407
Rural				0.229	(0.201)	0.253
Age				−0.738	(2.461)	0.764
Age_sq				0.109	(0.312)	0.726
Male				0.523	(0.608)	0.389
/Cut 1				1.481	(5.513)	0.788
/Cut 2				4.548	(5.517)	0.410
Observations	472			472		
Province FE	Yes			Yes		
Pseudo R2	0.0577			0.0893		

Standard errors in parentheses. ***p < 0.01, **p < 0.05, *p < 0.1.

The regression results of control variables in Model 2 (Table 3) are generally consistent with findings from existing literature. First, a significant positive correlation is observed between income and subjective well-being among disabled veterans—those with higher incomes tend to report greater well-being. This aligns with the findings of Zhang et al. (2024), who highlighted the positive impact of subsidies on the welfare of disabled veterans. Second, individuals with a medium level of education (e.g., high school or vocational college) exhibit higher levels of well-being compared to those with lower educational attainment. This outcome may be attributed to mechanisms related to social capital and employment transitions, as also suggested by Lester et al. (2022), who found that veterans with such educational backgrounds tend to report greater well-being. Regarding marital status, there are no significant differences in well-being between married individuals and those in other categories. Party membership and years of service both show positive but statistically insignificant effects, suggesting that political affiliation and service duration have limited influence on life satisfaction. The coefficient for rural hukou is also positive but not significant. Although the coefficients for age and its squared term indicate a mild inverted U-shaped relationship, neither is statistically significant. Lastly, no notable gender differences are found, which is largely due to the fact that the majority of respondents in the sample are male.

### 4.3 Robustness check

To ensure the robustness of the baseline findings, several alternative estimation strategies were employed, as presented in Table 4. Column (1) incorporates clustered robust standard errors into the baseline model to address potential intra-group correlation. The positive association between the breadth of social support networks (network) and subjective well-being remains statistically significant, confirming the robustness of the core result. In Column (2), the dependent variable is replaced with respondents’ self-rated mental state (happiness), which is recoded on a three-point scale: very poor = 1, average = 2, very good = 3—consistent with the coding of the original well-being variable. The regression results indicate that the coefficient of network remains significantly positive (*β* = 0.147, *p* < 0.01), suggesting that broader social support networks are positively associated not only with subjective well-being but also with individual mental status. Column (3) replaces the main explanatory variable with the natural logarithm of the number of WeChat contacts in the respondent’s mobile phone plus one (wechat). The estimation result shows that wechat is significantly positively correlated with subjective well-being (*β* = 0.164, *p* < 0.01), providing additional evidence from an alternative dimension to validate the main findings. In sum, the use of alternative outcome variables and estimation strategies consistently supports the core finding that the breadth of social support networks is positively associated with subjective well-being, further reinforcing the robustness of the study’s conclusions.

**Table 4:** Robustness test results.

Variables	Model 1			Model 2			Model 3		
	Well-being			Happiness			Well-being		
	Coef.	SE	*p*	Coef.	SE	p	Coef.	SE	*p*
Network	0.183***	(0.051)	0.000	0.147***	(0.051)	0.004			
Wechat							0.164***	(0.047)	0.001
Party_member	0.192	(0.129)	0.136	0.330	(0.269)	0.221	0.142	(0.265)	0.593
Service_time	0.003	(0.023)	0.877	−0.007	(0.021)	0.735	0.008	(0.021)	0.700
Income	0.348***	(0.072)	0.000	0.239	(0.169)	0.158	0.313*	(0.171)	0.067
Marital status (reference group: married)									
2.Marriage	−0.391	(0.404)	0.333	−1.293***	(0.448)	0.004	−0.417	(0.456)	0.361
3.Marriage	−0.483	(0.318)	0.128	−0.477	(0.388)	0.220	−0.549	(0.375)	0.143
Education level (reference group: low education)									
2.Education	0.514*	(0.298)	0.085	0.728***	(0.271)	0.007	0.504*	(0.258)	0.050
3.Education	−0.204	(0.270)	0.450	−0.332	(0.313)	0.289	−0.177	(0.309)	0.566
Military branch (reference group: other)									
1.Branch	−0.379	(0.626)	0.545	−0.918**	(0.414)	0.027	−0.386	(0.419)	0.356
2.Branch	−0.210	(0.514)	0.683	−0.270	(0.330)	0.413	−0.231	(0.336)	0.492
3.Branch	−0.682	(0.756)	0.367	−0.321	(0.459)	0.485	−0.721	(0.457)	0.114
4.Branch	0.322	(0.574)	0.575	0.429	(0.404)	0.289	0.281	(0.389)	0.470
Rural	0.229	(0.268)	0.393	0.374*	(0.208)	0.072	0.257	(0.201)	0.201
Age	−0.738	(1.167)	0.527	−0.281	(2.438)	0.908	−0.515	(2.451)	0.834
Age_sq	0.109	(0.126)	0.388	−0.068	(0.307)	0.824	0.111	(0.311)	0.722
Male	0.523**	(0.227)	0.021	0.828	(0.633)	0.191	0.557	(0.607)	0.359
/Cut1	1.481	(3.721)	0.691	−1.279	(5.535)	0.817	1.913	(5.497)	0.728
/Cut2	4.548	(3.768)	0.227	1.571	(5.536)	0.777	4.975	(5.502)	0.366
Observations	472			472			472		
Province FE	Yes			Yes			Yes		
Pseudo R2	0.0893			0.113			0.0884		

Standard errors in parentheses. ***p < 0.01, **p < 0.05, *p < 0.1.

### 4.4 Mediation effect test

This study introduces two mediating variables—perceived effectiveness of government assistance (institutional resource utilization) and trust among fellow veterans (relational social capital)—to examine the mechanisms linking social support network breadth and subjective well-being. Specifically, the perceived effectiveness of assistance from the Veterans Affairs Department (effective) measures the respondent’s subjective satisfaction with the outcome of seeking help from the department due to personal or family difficulties. This variable reflects the perceived efficacy of institutional support and serves as a key component of institutional social capital. It is measured as an ordinal categorical variable ranging from 1 to 3, with higher values indicating greater satisfaction: 1 = “problems unresolved, very poor effect,” 2 = “some problems resolved, moderate effect,” and 3 = “problems resolved, very good effect.” Trust among comrades (trust) captures the respondent’s perceived level of interpersonal trust within the veteran community, reflecting the strength of relational social capital. It is measured on a subjective 10-point scale, where 1 represents “complete distrust” and 10 indicates “complete trust.” This variable emphasizes emotional attributes and homogeneity, aiming to assess veterans’ sense of psychological security and mutual support within peer networks.

#### 4.4.1 Mediation effect of perceived government assistance

Table 5 examines the mediating role of perceived government assistance effectiveness (effective) in the relationship between social support network breadth (network) and subjective well-being. Model (1) shows a significant positive association between network breadth and perceived effectiveness of government assistance (*β* = 0.139, *p* < 0.01), indicating that disabled veterans with broader social connections are more likely to give higher evaluations of governmental support services. Model (2) demonstrates a significant positive relationship between assistance effectiveness and subjective well-being (*β* = 0.393, *p* < 0.01), suggesting that higher-quality institutional support significantly enhances veterans’ well-being. Model (3), which includes both variables simultaneously, shows that both network breadth and assistance effectiveness remain significantly associated with subjective well-being, indicating a partial mediation effect. Thus, Hypothesis H2a is supported.

**Table 5:** Mediation analysis of the effectiveness of government assistance.

Variables	(1)			(2)			(3)		
	Effective			Well-being			Well-being		
	Coef.	SE	*p*	Coef.	SE	*p*	Coef.	SE	*p*
Effective				0.393***	(0.104)	0.000	0.363***	(0.105)	0.001
Network	0.139***	(0.051)	0.006				0.167***	(0.051)	0.001
Party_member	0.220	(0.271)	0.418	0.126	(0.265)	0.635	0.167	(0.267)	0.532
Service_time	−0.041**	(0.021)	0.045	0.011	(0.021)	0.580	0.009	(0.021)	0.660
Income	−0.148	(0.171)	0.386	0.471***	(0.167)	0.005	0.378**	(0.170)	0.026
Marital status (reference group: married)									
2.Marriage	−0.033	(0.449)	0.942	−0.549	(0.455)	0.227	−0.400	(0.460)	0.385
3.Marriage	0.796**	(0.375)	0.034	−0.691*	(0.378)	0.067	−0.633*	(0.378)	0.094
Education level (reference group: low education)									
2.Education	−0.547**	(0.255)	0.032	0.657**	(0.261)	0.012	0.636**	(0.262)	0.015
3.Education	−0.100	(0.310)	0.746	−0.120	(0.310)	0.699	−0.170	(0.312)	0.586
Military branch (reference group: other)									
1.Branch	−0.295	(0.417)	0.479	−0.372	(0.420)	0.377	−0.338	(0.421)	0.422
2.Branch	0.400	(0.327)	0.221	−0.289	(0.339)	0.393	−0.276	(0.340)	0.418
3.Branch	0.515	(0.451)	0.254	−0.806*	(0.459)	0.079	−0.772*	(0.462)	0.095
4.Branch	0.181	(0.385)	0.639	0.196	(0.390)	0.615	0.282	(0.393)	0.474
Rural	−0.042	(0.202)	0.834	0.222	(0.201)	0.269	0.231	(0.202)	0.252
Age	6.692	(5.360)	0.212	−0.951	(2.469)	0.700	−1.692	(2.491)	0.497
Age_sq	−0.764	(0.694)	0.271	0.100	(0.311)	0.748	0.214	(0.315)	0.496
Male	−0.242	(0.645)	0.708	0.602	(0.608)	0.322	0.587	(0.617)	0.341
/Cut1	13.726	(10.594)	0.195	1.721	(5.510)	0.755	0.207	(5.553)	0.970
/Cut2	14.114	(10.595)	0.183	4.790	(5.515)	0.385	3.336	(5.556)	0.548
Observations	472			472			472		
Province FE	Yes			Yes			Yes		
Pseudo R2	0.0618			0.0909			0.103		

Standard errors in parentheses. ***p < 0.01, **p < 0.05, *p < 0.1.

#### 4.4.2 Mediation effect of peer trust

Table 6 tests the mediating role of comrade trust (trust) in the relationship between social support network breadth (network) and subjective well-being. Model (1) reveals a significant positive association between network breadth and comrade trust (*β* = 0.160, *p* < 0.05), suggesting that veterans with broader social connections tend to report higher levels of trust among comrades. Model (2) shows that comrade trust is significantly and positively associated with subjective well-being (*β* = 0.221, *p* < 0.01), indicating a potential link between informal relational resources and veterans’ well-being. Model (3) includes both network breadth and comrade trust, and both remain significantly associated with well-being. This suggests that comrade trust partially mediates the relationship between network breadth and subjective well-being, thus supporting Hypothesis H2b.

**Table 6:** Mediation analysis of peer trust.

Variables	(1)			(2)			(3)		
	Trust			Well-being			Well-being		
	Coef.	SE	*p*	Coef.	SE	*p*	Coef.	SE	*p*
Trust				0.221***	(0.054)	0.000	0.199***	(0.055)	0.000
Network	0.160**	(0.076)	0.034				0.158***	(0.051)	0.002
Party_member	0.157	(0.367)	0.669	0.101	(0.266)	0.704	0.146	(0.267)	0.585
Service_time	−0.028	(0.030)	0.349	0.008	(0.021)	0.688	0.006	(0.021)	0.760
Income	−0.182	(0.228)	0.425	0.455***	(0.168)	0.007	0.365**	(0.171)	0.033
Marital status (reference group: married)									
2.Marriage	−0.852	(0.655)	0.194	−0.428	(0.458)	0.350	−0.303	(0.462)	0.512
3.Marriage	−0.703	(0.501)	0.160	−0.481	(0.376)	0.201	−0.454	(0.376)	0.228
Education level (reference group: low education)									
2.Education	−0.343	(0.357)	0.336	0.590**	(0.259)	0.023	0.564**	(0.260)	0.030
3.Education	0.475	(0.423)	0.262	−0.161	(0.308)	0.601	−0.211	(0.311)	0.496
Military branch (reference group: other)									
1.Branch	0.807	(0.617)	0.191	−0.566	(0.426)	0.184	−0.518	(0.426)	0.224
2.Branch	0.083	(0.482)	0.864	−0.276	(0.336)	0.413	−0.262	(0.338)	0.438
3.Branch	−0.081	(0.650)	0.901	−0.723	(0.458)	0.114	−0.706	(0.461)	0.126
4.Branch	−0.300	(0.543)	0.581	0.238	(0.387)	0.538	0.316	(0.390)	0.418
Rural	−0.106	(0.276)	0.702	0.281	(0.201)	0.163	0.278	(0.202)	0.169
Age	−11.314	(13.012)	0.385	0.097	(2.461)	0.969	−0.719	(2.485)	0.772
Age_sq	1.532	(1.688)	0.364	−0.026	(0.311)	0.933	0.094	(0.315)	0.764
Male	1.425	(1.530)	0.352	0.524	(0.604)	0.386	0.512	(0.613)	0.403
/Cut1	−3.316	(1,407.996)	0.998	3.253	(5.511)	0.555	1.553	(5.561)	0.780
/Cut2	−3.214	(1,407.996)	0.998	6.354	(5.519)	0.250	4.703	(5.565)	0.398
/Cut3	−3.029	(1,407.996)	0.998						
/Cut4	−2.987	(1,407.996)	0.998						
/Cut5	−2.717	(1,407.996)	0.998						
/Cut6	−2.138	(1,407.996)	0.999						
/Cut7	−1.935	(1,407.996)	0.999						
/Cut8	−0.944	(1,407.996)	0.999						
/Cut9	−0.030	(1,407.996)	1.000						
Observations	472			472			472		
Province FE	Yes			Yes			Yes		
Pseudo R2	0.384			0.0941			0.105		

Standard errors in parentheses. ***p < 0.01, **p < 0.05, *p < 0.1.

#### 4.4.3 Mediation analysis based on the bootstrap method

To further assess the robustness of the previously identified mediation pathways, this study employed the bootstrap method to examine the indirect effects of the two mediators—perceived effectiveness of government assistance and comrade trust. A total of 500 resampling iterations were conducted, and bias-corrected confidence intervals (CIs) were constructed. As shown in Table 7, social support network breadth exerted significant indirect effects on subjective well-being through both mediators, with the 95% CIs of the indirect effects excluding zero. In both models, the direct effects remained statistically significant, indicating that both perceived government assistance effectiveness and comrade trust serve as partial mediators in the relationship. These findings further validate the dual-pathway mechanism by which *formal institutional resources* and *informal emotional resources* jointly enhance veterans’ well-being, confirming the critical role of institutional support and interpersonal trust as core components of social capital.

**Table 7:** Mediation analysis based on the bootstrap method.

Trails	Model	Efficiency value	Standard error	95% confidence interval	
				Lower limit	Higher limit
Network →effective→ well-being	Direct effect	0.047	0.017	0.014	0.079
	Indirect effect	0.008	0.004	0.000	0.016
Network →trust→ well-being	Direct effect	0.049	0.016	0.016	0.081
	Indirect effect	0.006	0.003	0.001	0.012

### 4.5 Moderation effect test

To examine the moderating effect of institutional resource awareness on policy effectiveness, this study introduces the variable of veterans’ *policy awareness* (awareness). This variable captures respondents’ familiarity with core policies related to veterans—such as the *Law on the Protection of Veterans’ Rights and Interests*, the *Resettlement Regulation*, and the *Preferential Treatment Regulation*. It serves as a key indicator of *institutional information capital*. The variable is coded as an ordinal categorical variable ranging from 1 to 3: 1 = “completely unaware,” 2 = “aware,” and 3 = “very well informed.” Higher values indicate a more comprehensive understanding of policy content, procedures, and associated rights and obligations, thus reflecting a stronger capacity to navigate institutional structures.

The regression results in Table 8 show that *social support network breadth* (network) is significantly and positively associated with the *effectiveness of government assistance* (effective) (Coef. = 0.435, *p* < 0.001), suggesting that veterans with broader social networks are more likely to report better assistance outcomes. *Policy awareness* (awareness) is also significantly and positively associated with assistance effectiveness (Coef. = 1.828, *p* < 0.001), indicating that veterans with higher policy awareness perceive government assistance to be more effective.

**Table 8:** Moderation analysis.

Variables	(1)		
	Effective		
	Coef.	SE	*p*
Network	0.435***	(0.099)	0.000
Awareness	1.828***	(0.366)	0.000
Network_awareness	−0.270***	(0.078)	0.001
Party_member	0.027	(0.271)	0.920
Service_time	0.007	(0.021)	0.744
Income	0.323*	(0.171)	0.059
Marital status (reference group: married)			
2.Marriage	−0.157	(0.471)	0.739
3.Marriage	−0.486	(0.379)	0.200
Education Level (reference group: low education)			
2.Education	0.575**	(0.261)	0.028
3.Education	−0.176	(0.314)	0.575
Military branch (reference group: other)			
1.Branch	−0.230	(0.424)	0.588
2.Branch	−0.149	(0.340)	0.662
3.Branch	−0.563	(0.463)	0.224
4.Branch	0.353	(0.393)	0.369
Rural	0.193	(0.204)	0.344
Age	−0.798	(2.527)	0.752
Age_sq	0.102	(0.320)	0.750
Male	0.730	(0.610)	0.231
/Cut1	2.608	(5.681)	0.646
/Cut2	5.855	(5.687)	0.303
Observations	472		
Province FE	Yes		
Pseudo R2	0.124		

Standard errors in parentheses, ***p < 0.01, **p < 0.05, *p < 0.1.

A further examination of the moderation effect reveals that the interaction term between the breadth of social support networks and policy awareness (*network_awareness*) is negative and statistically significant (Coef. = − 0.270, *p* < 0.01). This indicates a negative moderating effect of policy awareness on the relationship between social support network breadth and aid effectiveness. Specifically, as policy awareness increases, the positive impact of social support networks on aid effectiveness diminishes. This may suggest that veterans with higher levels of policy awareness are less reliant on social support networks, or that policy awareness partially substitutes for the function of such networks. In summary, policy awareness not only directly enhances aid effectiveness but also moderates the relationship between social support networks and aid outcomes. This underscores the critical role of institutional resource cognition in improving veterans’ welfare and supports Hypothesis H3.

### 4.6 Heterogeneity analysis

#### 4.6.1 Heterogeneity analysis by disability severity

To further examine the heterogeneity in the impact of social support networks on subjective well-being, this study conducts a subgroup analysis based on disability severity among veterans. According to the Disability Rating Standards for Military Personnel, disability levels (*degree*) range from 1 to 10, with higher values indicating greater severity. The sample is divided into two subgroups: Group I includes veterans with mild disabilities (levels 1–5), and Group II comprises those with severe disabilities (levels 6–10). This classification aims to control for the potential confounding effect of physical health on well-being.

Table 9 presents the results of subgroup analysis by disability severity. Social support network breadth (network) is positively and significantly associated with subjective well-being in both groups, although the strength of this relationship differs. Among veterans with mild disabilities (Group I), the coefficient for network is 0.436 and statistically significant at the 1% level (*p* = 0.001), suggesting a strong potential association between broader social connections and well-being. In contrast, for veterans with severe disabilities (Group II), the coefficient is smaller (0.125) but remains significant at the 5% level (*p* = 0.027), indicating that the positive association between social networks and well-being persists, albeit with weaker intensity.

**Table 9:** Heterogeneity analysis: the impact of social support networks on subjective well-being across groups with different disability levels.

Variables	(1)			(2)		
	Well-being			Well-being		
	Disability degree group I (mild disability: levels 1–5)			Disability degree group ii (severe disability: levels 6–10)		
	Coef.	SE	p	Coef.	SE	p
Network	0.436***	(0.137)	0.001	0.125**	(0.057)	0.027
Party_member	−0.719	(0.589)	0.222	0.372	(0.317)	0.241
Service_time	−0.003	(0.064)	0.961	0.002	(0.023)	0.940
Income	0.888***	(0.344)	0.010	0.302	(0.213)	0.155
Marital status (reference group: married)						
2.Marriage	0.976	(0.965)	0.312	−0.537	(0.571)	0.347
3.marriage	−4.171**	(2.029)	0.040	−0.267	(0.410)	0.516
Education level (reference group: low education)						
2.Education	−0.267	(0.808)	0.741	0.633**	(0.285)	0.027
3.Education	−0.167	(0.903)	0.854	−0.029	(0.343)	0.933
Military branch (reference group: other)						
1.Branch	−0.802	(0.947)	0.397	−0.124	(0.494)	0.802
2.Branch	−0.028	(0.813)	0.972	−0.361	(0.381)	0.343
3.Branch	−0.017	(1.105)	0.988	−0.732	(0.526)	0.165
4.Branch	0.170	(1.012)	0.867	0.422	(0.437)	0.334
Rural	1.153**	(0.532)	0.030	0.033	(0.232)	0.886
Age	−65.684**	(31.411)	0.037	−0.143	(2.562)	0.955
Age_sq	8.445**	(3.985)	0.034	0.027	(0.323)	0.934
/Cut1	−119.265*	(61.000)	0.051	1.850	(6.001)	0.758
/Cut2	−115.393*	(60.924)	0.058	5.017	(6.005)	0.403
Male				0.614	(0.628)	0.329
Observations	107			365		
Province FE	Yes			Yes		
Pseudo R2	0.222			0.112		
Chow Test	χ^2^ = 3.82			*p* = 0.0508		

Standard errors in parentheses. ***p < 0.01, **p < 0.05, *p < 0.1.

Furthermore, the test for coefficient difference between groups yields χ^2^ = 3.82, *p* = 0.0508, indicating a statistically significant disparity in the effect of social networks on well-being across the two subgroups. This suggests that the marginal utility of social capital is greater for mildly disabled veterans. From the perspective of resource access, these individuals are more capable of actively mobilizing their networks and maintaining higher levels of participation in social interactions, thereby enhancing their well-being. In contrast, veterans with severe disabilities face multiple barriers—including physical limitations, reduced mobility, and increased dependency—that hinder their ability to access and utilize existing social networks effectively (Rapegno and Ravaud, 2017; Klenk et al., 2019). These constraints not only reduce the frequency and quality of social engagement, but also intensify feelings of isolation and helplessness, diminishing the positive psychological effects of social support. Moreover, institutional barriers and reduced communication capacity may further limit their ability to recognize and leverage available social resources. Compared with their mildly disabled counterparts, severely disabled veterans are more likely to fall into a structural dilemma characterized by high need but low access, which reduces the efficiency of social capital conversion.

In summary, while social support networks positively influence well-being across all levels of disability, the strength of this effect is moderated by physical health status. This finding underscores the need for differentiated social support interventions tailored to the degree of disability in efforts to promote the social integration of veterans.

#### 4.6.2 Heterogeneity analysis by age differences

To examine the age heterogeneity in the impact of social support networks on subjective well-being, this study conducts a subgroup analysis based on age, using 60 years as the dividing line. The age of 60 serves not only as a common benchmark for statutory retirement in China but also marks a critical transition into old age for military veterans, carrying significant practical and policy implications. Therefore, using 60 as the threshold helps identify differences in how social support affects well-being across life stages.

Table 10 reports the empirical results by age group. Among veterans aged 60 and below, the coefficient of network on subjective well-being is 0.151 and statistically significant at the 1% level (*p* = 0.008), indicating a significant positive association between the breadth of social support networks and well-being in the younger cohort. For those aged above 60, the coefficient increases to 0.431 with stronger statistical significance (*p* = 0.002), suggesting that social connections exert a more substantial and pronounced effect on well-being among older veterans.

**Table 10:** Heterogeneity analysis: the impact of social support networks on subjective well-being across different age groups.

Variables	(1)			(2)		
	Effective			Well-being		
	Age ≤ 60 years			Age > 60 years		
	Coef.	SE	*p*	Coef.	SE	*p*
Network	0.151***	(0.057)	0.008	0.431***	(0.138)	0.002
Party_member	0.243	(0.321)	0.449	0.513	(0.577)	0.374
Service_time	0.007	(0.023)	0.756	0.024	(0.066)	0.712
Income	0.114	(0.217)	0.600	0.944***	(0.365)	0.010
Marital status (reference group: married)						
2.Marriage	−0.398	(0.511)	0.437	−0.865	(1.313)	0.510
3.Marriage	−0.596	(0.423)	0.159	−3.222*	(1.652)	0.051
Education level (reference group: low education)						
2.Education	0.606**	(0.281)	0.031	0.917	(0.891)	0.303
3.Education	−0.033	(0.333)	0.921	0.793	(1.222)	0.516
Military branch (reference group: other)						
1.Branch	−0.804	(0.498)	0.106	0.629	(1.008)	0.533
2.Branch	−0.698*	(0.418)	0.095	1.034	(0.695)	0.137
3.Branch	−1.314**	(0.548)	0.017	2.307**	(1.044)	0.027
4.Branch	−0.113	(0.451)	0.802	0.724	(2.197)	0.742
Rural	0.079	(0.229)	0.730	0.890*	(0.527)	0.091
Age	−25.678	(15.985)	0.108	1.906	(6.292)	0.762
Age_sq	3.465	(2.158)	0.108	−0.282	(0.476)	0.553
Male	0.833	(0.713)	0.242	−0.785	(1.561)	0.615
/Cut1	−47.014	(29.841)	0.115	11.459	(21.002)	0.585
/Cut2	−44.090	(29.828)	0.139	15.868	(21.073)	0.451
Observations	362			110		
Province FE	Yes			Yes		
Pseudo R2	0.0921			0.232		
Chow test	χ^2^ = 2.73			*p* = 0.0983		

Standard errors in parentheses. ***p < 0.01, **p < 0.05, *p < 0.1.

The test for group differences yields a borderline significant result (χ^2^ = 2.73, *p* = 0.0983), implying some heterogeneity in the association between social networks and well-being across age groups. This difference may stem from age-related changes in lifestyle and psychological needs. On one hand, older veterans tend to experience reduced physical capacity and a narrower range of daily activities, which limits opportunities for social participation and increases the likelihood of loneliness and social isolation (Shaw et al., 2007; Cornwell and Waite, 2009). As a result, they may rely more heavily on external social connections to maintain a sense of well-being. On the other hand, compared with younger individuals, older adults—especially after withdrawing from the workforce—derive their primary social roles from close informal networks such as family and neighbors, where the emotional function of social support becomes more critical (Kauppi et al., 2021). In sum, although social connections benefit veterans across age groups, their impact is more pronounced among older individuals.

In summary, social support networks are a vital resource for enhancing the subjective well-being of veterans, with varying degrees of reliance across age groups. Policy development should pay particular attention to the unique needs of older veterans in maintaining social relationships, and strengthen mechanisms for social integration to further improve their quality of life in later years.

## 5 Conclusion

Based on a sample of 472 disabled military veterans in China, this study systematically investigates the mechanisms through which the breadth of social support networks influences subjective well-being. The key findings are as follows:

First, the expansion of social support networks is positively associated with enhanced subjective well-being among disabled veterans. Although previous studies have noted that broad social connections can help alleviate psychological distress and social isolation during the transition to civilian life (Demers, 2011), most have not focused on disabled veterans as a distinct group. This study not only empirically tests the applicability of this mechanism in this specific population but also enhances the credibility and generalizability of the findings through robustness checks and variable substitution, thereby expanding the theoretical scope of existing literature.

Second, the perceived effectiveness of government assistance and trust among fellow veterans serve as significant dual mediators between structural social capital and subjective well-being, revealing a synergistic embedding mechanism between institutional and relational resources. This finding empirically supports Coleman’s (1990a,b) theory of the multidimensional nature of social capital—that it exists both in informal networks and within formal institutional arrangements. It also aligns with Krishna’s (2002) definition of the functional and structural duality of social capital. While existing studies often focus on generalized institutional or social trust, this study identifies the unique emotional trust mechanism of “comrade trust” among disabled veterans, thus extending Nahapiet and Ghoshal’s (1998) concept of “relational embeddedness.” Specifically, the shared service experience not only enhances internal group cohesion but also amplifies the psychological benefits of social capital, enriching the application of social capital theory in special populations.

Third, the relationship between social support networks and subjective well-being is moderated by policy awareness, showing a marginal substitutive effect. This finding validates and extends Putnam et al.’s (1994) perspective on the substitutability of social resources. When one type of resource is sufficiently available, dependence on another may decrease (Huang et al., 2025), meaning that individuals can draw on alternative social resources to meet their needs. When formal institutional informational capital is high, the marginal utility of informal networks diminishes, reflecting a dynamic interplay of complementarity and substitution between formal and informal forms of social capital.

Fourth, heterogeneity analysis shows that the positive association between social support networks and well-being is more pronounced among elderly veterans and those with mild disabilities. This aligns with findings by Cornwell and Waite (2009) on emotional needs in older populations and supports Krishna’s (2002) observation that limited physical functioning reduces the utility of social networks. Life stage and physical condition significantly shape individuals’ ability to perceive and utilize social capital, suggesting that policies should account for group heterogeneity to effectively enhance access to social resources.

In summary, this study confirms the applicability of social capital theory to the special population of disabled military veterans and, through the inclusion of mediating and moderating mechanisms, reveals the dynamic interactions between institutional and relational social capital in fostering subjective well-being. These findings extend the explanatory power of resource substitution theory and resilience theory among disadvantaged groups, highlighting the need for coordinated consideration of both formal institutional support and informal relational support in policy design and social work practice aimed at improving psychological well-being and promoting the social integration of disabled veterans.

Based on these key findings, the following policy recommendations are proposed:

First, it is crucial to expand veterans’ social support networks and strengthen their capacity to access social capital. An increase in the breadth of social support networks can significantly improve the subjective well-being of disabled veterans. Therefore, relevant government agencies and community organizations should actively develop social support networks tailored for disabled veterans. Specific measures include: First, establish dedicated social platforms and service networks for veterans, encouraging disabled veterans to actively participate in various community and social organization activities to enhance their social engagement and facilitate effective social integration. Second, encourage, support, and guide various volunteer service organizations to establish long-term relationships with veteran groups by conducting mutual aid, assistance, and emotional exchange activities, thereby broadening their social connections. Third, optimize the functional design of existing veteran service centers by adding interactive programs that expand social networks, such as social integration workshops and networking events. This systematic approach will improve veterans’ capacity to acquire social capital, ultimately promoting their mental health and subjective well-being.

Second, it is important to enhance the precision of government institutional responses and strengthen the perceived accessibility of institutional resources. Government assistance effectiveness plays a significant mediating role between social support networks and subjective well-being. Therefore, government agencies should further optimize the precision and effectiveness of institutional services for veterans, especially disabled veterans. The specific measures are as follows: First, improve the professional competence and service awareness of personnel in veteran affairs departments, establish regular follow-up visits and service effectiveness evaluation mechanisms to ensure timely policy implementation and prompt feedback handling; second, set up a rapid response mechanism to simplify the channels for veterans to report demands and difficulties, shorten the problem resolution cycle, and enhance the perceived accessibility of institutional resources; third, utilize “digitalization + artificial intelligence” tools to build a real-time tracking and evaluation system for veteran institutional support, increasing transparency and openness in veteran affairs management, thereby reinforcing the critical role of policy services in improving the subjective well-being of disabled veterans.

Third, it is essential to enhance policy awareness and strengthen the institutional system for information dissemination. Policy awareness significantly moderates the impact of social support networks on the subjective well-being of disabled veterans. To this end, efforts should focus on strengthening the promotion and dissemination of veteran-related policies to improve their policy awareness. Specifically, three measures are recommended: First, leverage new media platforms, official WeChat public accounts, and mini-programs to regularly deliver policy interpretations and implementation guidelines related to veterans, thereby establishing a systematic and normalized policy communication mechanism. Second, implement targeted and stratified communication strategies based on veterans’ educational backgrounds, ages, and disability levels to ensure effective reception and comprehension of policy information within this group. Third, establish a policy lecturer system at the community level, where trained volunteers or veteran representatives conduct regular policy briefings. This approach not only reduces the cost of information dissemination but also enhances the accessibility and transparency of policies, ultimately improving veterans’ sense of policy acquisition and overall well-being.

Finally, it is necessary to implement differentiated intervention strategies tailored to the heterogeneous needs of specific groups. Among elderly veterans and those with lower levels of disability, the positive impact of social support networks on subjective well-being is more pronounced. Therefore, relevant government agencies should further consider the heterogeneity of group needs when formulating and implementing veteran assistance policies and services. Specifically: First, for elderly veterans, address their practical needs by enhancing the construction of social support networks, such as establishing neighborly-friendly communities, providing emotional companionship services, and organizing various interest groups to alleviate loneliness and social isolation during old age. Second, for veterans with lower disability levels, strengthen support for social integration and employment promotion by increasing opportunities for vocational retraining, expanding flexible employment channels, and offering psychological counseling services to facilitate role transition and social inclusion. Third, establish a tiered and classified mental health intervention system that regularly conducts psychological assessments and provides mental health services tailored to the needs of different disability levels and age groups, thereby effectively improving psychological resilience and subjective well-being across diverse populations.

## Data Availability

The datasets presented in this article are not readily available because participants did not provide explicit consent for their data to be shared beyond the research team, in accordance with ethical guidelines on confidentiality. Requests to access the datasets should be directed to Yu Cao, caoyu2004020906@163.com.
