# Supplementary material for: Social support networks and well-being of disabled veterans: the dual roles of institutional support and peer trust
Source: Front Psychol. 2025 Aug 22;16:1654845. doi: 10.3389/fpsyg.2025.1654845 (PMC12412134; doi:10.3389/fpsyg.2025.1654845)
Supplement: Supplementary file 1 [file Supplementary_file_1.docx]

**Appendix I:**

**Survey Questionnaire for Disabled Veterans**

**1 What is your gender?**

1. Male
2. Female

**2 Date of birth:** Year____ Month____

**3 Date of enlistment:** Year____ Month____

**4 Date of discharge:** Year____ Month____

**5 Which branch of the military did you serve in at the time of enlistment?**

1. Navy
2. Army (including former Railway Corps, Engineering Corps, etc.)
3. Air Force
4. Armed Police
5. Rocket Force
6. Strategic Support Force
7. Nuclear-related or nuclear test units
8. Other (please specify):

**6 Are you a member of the Communist Party of China (CPC) ?**

1. No, I am a member of the general public (including Communist Youth League members)
2. Yes. I joined the Party in: Year____ Month____ (enter the date you became a probationary CPC member)
3. No, but I am a member of a democratic party

**7 What is your highest level of education?**

1. No formal education
2. Primary school
3. Junior high school
4. Senior high school / Secondary technical school / Vocational school
5. Full-time associate degree
6. Part-time associate degree
7. Full-time bachelor’s degree
8. Part-time bachelor’s degree
9. Full-time master’s degree
10. Part-time master’s degree
11. Doctoral degree

**8 What is your current marital status?**

1. Married
2. Unmarried
3. Divorced
4. Widowed

**9 What is the nature of your current registered residence?**

1. Rural
2. Urban

**10 What was your income in 2022? (in 10,000 yuan)**

**11 Since the COVID-19 pandemic, how many hours do you work on average per day in 2023?**

1. 8 hours or less
2. 9 hours
3. 10 hours
4. 11 hours
5. 12 hours or more

**12 What is the floor area of your current residence? square meters**

**13 Which of the following best describes your current housing situation?**

1. Self-built rural housing
2. Owned urban house (commercial house, housing reform house, economically affordable house, or equivalent)
3. Government-subsidized low-rent/public rental housing
4. Collective dormitory provided by the employer after demobilization
5. Living in a relative’s or friend’s home
6. Renting on the open market
7. Temporary housing provided by the government due to demolition
8. Military-assigned housing
9. Other (please specify):
10. Not sure

**14 Are you currently living in this residence?**

1. Yes, I live in my own residence
2. No, I do not live in my own residence

**15 Have you received any military honors or awards?**

| **Award Category** | **Number of Awards**  **(enter 0 if none)** |
| --- | --- |
| **Commendation**  **(including Outstanding Soldier)** |  |
| **Third-Class Merit** |  |
| **Second-Class Merit** |  |
| **First-Class Merit** |  |
| **Military Region-Level Honorary Title or Equivalent** |  |
| **Other (please specify):** | |

**16 Are you a person with a disability?**

1. Yes. Disability level: Level
2. No

**17 Are you a military service-related disabled veteran?**

1. Yes. Military disability level: Level
2. No

**18 What is your current employment status?**

1. Stably employed (defined as having at least three types of social insurance coverage)
2. Unstably employed (e.g., gig work, rideshare driving, courier services)
3. Unemployed or laid off
4. Returned to school
5. Retired
6. Other (please specify):

**19 What is the amount of your annual pension or compensation? （in yuan）**

**20 Are you able to receive your pension or compensation on time? (Including disabled veterans and families of fallen soldiers)**

1. I am not an entitled recipient, so this question does not apply to me.
2. Yes, I receive my pension on time. Please specify your category of entitled recipient:
3. No, I do not receive my pension on time. Please specify your category of entitled recipient:

**21 Are you receiving the full amount of your entitled pension or compensation? (Including disabled veterans and families of fallen soldiers)**

1. I am not an entitled recipient, so this question does not apply to me
2. Yes, I receive the full amount
3. No, I do not receive the full amount

**22 How would you describe your current overall life and work satisfaction?**

1. I feel very satisfied with my life and work.
2. I feel somewhat satisfied with my life and work.
3. I feel very dissatisfied with my life and work.

**23 How many contacts do you have on your mobile phone? (     ); How many contacts do you have on WeChat? (     ); (You can check the number by scrolling to the bottom of your WeChat contact list)**

**24 Have you ever sought assistance from the Veterans Affairs Department due to personal or family difficulties? If so, how effective was the response?**

1. The issue was resolved, and the outcome was very good
2. The issue was partially resolved, and the outcome was average
3. The issue was not resolved, and the outcome was very poor

**25 Between April 2022 and May 2023, how many veteran reunions or gatherings did you attend?**

1. None
2. Once
3. 2 to 4 times
4. 5 times or more

**26 How familiar are you with policies related to veterans (e.g., Law on the Protection of Veterans, Resettlement Regulations, Preferential Treatment Regulations)?**

1. Very familiar
2. Somewhat familiar
3. Not familiar at all

**27 How would you evaluate your current mental and emotional well-being?**

1. Very good
2. Average
3. Very poor

**28 Please rate your level of trust in interpersonal relationships on a scale from 1 to 10, where 1 indicates complete distrust and 10 indicates complete trust:**

|  | Complete distrust Complete trust |
| --- | --- |
| Current level of trust between people | 1 2 3 4 5 6 7 8 9 10 |
| Current level of trust between fellow veterans | 1 2 3 4 5 6 7 8 9 10 |
